# Safety Evaluation of Cyhalofop-Butyl on Agronomic Traits and Antioxidant Enzyme Activities in Foxtail Millet

**DOI:** 10.3390/plants14203170

**Published:** 2025-10-15

**Authors:** Chunyan Hu, Tingting Chen, Xutao Zhai, Jingtao Yuan, Suqi Shang, Yinyuan Wen, Xi’e Song, Juan Zhao, Hui Cao, Shuqi Dong

**Affiliations:** 1 College of Plant Protection, Shanxi Agricultural University, Jinzhong 030800, China; hucy4216@sxau.edu.cn (C.H.); xutaozhai@163.com (X.Z.); yuanjingtao2025@163.com (J.Y.); 2 College of Agriculture, Shanxi Agricultural University, Jinzhong 030800, China; sxndctt@163.com (T.C.); ssq2025@163.com (S.S.); wenyinyuan@126.com (Y.W.); sxndsxe@163.com (X.S.); sxndzhaojuan@163.com (J.Z.); 3 Special Orphan Crops Research Center of the Loess Plateau, MARA, Shanxi Agricultural University, Jinzhong 030800, China

**Keywords:** cyhalofop-butyl, foxtail millet, agronomic traits, antioxidant enzyme activity, weed control efficacy

## Abstract

Weed infestation severely impairs foxtail millet growth, while new herbicide development faces long cycles and high costs. This study examined cyhalofop-butyl (effective against *Echinochloa crus-galli* (*E. crus-galli*) in paddy fields) to assess its effects on agronomic traits, antioxidant enzyme activities, malondialdehyde (MDA) content of Jingu 21, and weed control efficacy in foxtail millet fields, aiming to screen safe, effective concentrations. Results showed that sole cyhalofop-butyl inhibited foxtail millet growth: high-dose treatments retained significant agronomic trait inhibition 30 days post-spray. Early post-spray, superoxide dismutase (SOD) and peroxidase (POD) activities showed a “first increase then decrease” pattern, catalase (CAT) a “first decrease then increase” trend, and MDA content rose; index differences from the water control narrowed over time, though 90 g a.i./hm^2^ still caused higher leaf SOD activity and MDA content in later stages. For weed control, cyhalofop-butyl effectively controlled *E. crus-galli* (control effect “first increase then decrease,” 90 g a.i./hm^2^ optimal) and *Digitaria sanguinalis* (*D. sanguinalis*) (control effect rising over time) in foxtail millet fields. On the whole, 22.5 g a.i./hm^2^ and 45 g a.i./hm^2^ of cyhalofop-butyl are safe for Jingu 21, and 67.5 g a.i./hm^2^ is also a safe concentration, so 45–67.5 g a.i./hm^2^ can be preferred for comprehensive weed control.

## 1. Introduction

In recent years, chemical herbicides have been widely used in agricultural production. However, the long-term use of a single herbicide tends to enhance the herbicide resistance of weeds, making it difficult to effectively control weed infestation. The mixed use of herbicides, as a key means to optimize weed control schemes, can achieve multiple goals such as expanding the weed control spectrum, improving the weed control effect, delaying the development of weed resistance, reducing phytotoxicity, and lowering the risk of residues.

Cyhalofop-butyl is an inhibitor of acetyl-CoA carboxylase (ACCase, EC. 6.4.1.2), belonging to the HRAC group A, and a post-emergence herbicide used for weed control in paddy fields [1,2]. This herbicide is absorbed by the leaves and leaf sheaths of crops, translocated through the phloem, and accumulated in the meristematic regions. By inhibiting the activity of acetyl-CoA carboxylase (ACCase), it blocks the fatty acid synthesis process, leading to abnormal cell growth and division, damage to lipid-containing structures such as the membrane system, and ultimately the death of weeds [3]. In plant cells, ACCase mainly catalyzes the synthesis of malonyl-CoA from acetyl-CoA and bicarbonate. Malonyl-CoA is an important substrate for fatty acid synthesis in plastids, and the use of ACCase inhibitors inhibits fatty acid synthesis in plant plastids. Aryloxyphenoxypropionate herbicides can also cause sensitive plants to produce excessive reactive oxygen species (ROS), resulting in damage to the cell membrane, changes in membrane fluidity, loss of cell components, and ultimately cell death [4].

While herbicides control weeds, they may also cause phytotoxicity to crops, interfere with the physiological and biochemical metabolism of crops, and thereby affect the growth and development of crops. In actual production, if the herbicide dosage is too low, the weed control effect will be poor, and weeds cannot be effectively controlled. If the dosage is too high, it will exceed the tolerance limit of crops, causing severe phytotoxicity. Zhang et al. [5] found that there is a dose-effect relationship between herbicides and their weed control efficacy, that is, as the dosage increases, the weed control effect improves accordingly. However, an excessively high dosage may not only exceed the safe range of crops but also easily increase the risk of phytotoxicity due to uneven spraying. In addition, herbicides can also directly affect yield components by influencing the agronomic traits of crops, ultimately leading to yield reduction [6]. Previous research results have shown that after 30 days of exposure treatment, prometryne severely inhibits the growth of leguminous plants, specifically manifested as a significant reduction in plant height and leaf area, indicating that prometryne induces leguminous plants into a stress state [7]. Similar effects have also been observed in other species after prometryne treatment, such as wheat [8,9], peas [10], and beans [11].

Oxidative stress induced by herbicides leads to cell peroxidation and molecular damage through the excessive accumulation of reactive oxygen species (ROS) [12,13]. To inhibit the effects of ROS, plants have evolved protective enzymatic mechanisms, such as those involving SOD, POD, and CAT [14]. Studies have shown that the herbicide metolachlor can cause plants to produce a large amount of ROS and free radicals, reduce the structural and functional stability of the cell membrane, and lead to the excessive accumulation of MDA, a product of membrane lipid peroxidation [15]. In addition, the enzymatic reaction system of plants is quickly activated, and SOD and POD are important detoxification mechanisms in plants, which work together to immediately scavenge reactive oxygen species [16]. Among them, SOD is the main protective enzyme in the membrane lipid peroxidation defense system, which can directly react with O_2_^−^ in cells to generate H_2_O_2_ and O_2_^−^; CAT and POD mainly exist in the peroxisomes of plants, and their main function is to decompose H_2_O_2_, eliminating the damage of O_2_^−^ to organisms [17,18]. Wang and Zhou [19] believed that chlorsulfuron increases the activities of SOD and POD as well as the content of malondialdehyde (MDA) in wheat.

The seedling emergence stage of foxtail millet overlaps with the dry and rainless season in northern China, which limits the weed control effect of soil-applied herbicides. Moreover, there is a lack of foliar herbicides, making weed control more difficult. Manual weeding is costly and inefficient, while chemical weeding has the advantages of being economical and efficient. However, foxtail millet is relatively sensitive to herbicides, so screening suitable herbicides and their dosages has become a key issue. This study selects cyhalofop-butyl, which has excellent efficacy in controlling gramineous weeds such as *E.crus-galli* in paddy fields, and targets the conventional foxtail millet variety Jingu 21. Through setting up indoor pot experiments and field experiments with different dosages, it systematically evaluates the safety of cyhalofop-butyl on Jingu 21 and its weed control efficacy in foxtail millet fields, screens out the appropriate application dosage, provides theoretical support for the safe and efficient weed control in foxtail millet fields, contributes to the large-scale light and modern cultivation of foxtail millet, and ensures national food security.

## 2. Results

### 2.1. Effects of Spraying Different Doses of Cyhalofop-Butyl on Agronomic Traits of Foxtail Millet

**Figure 1:**
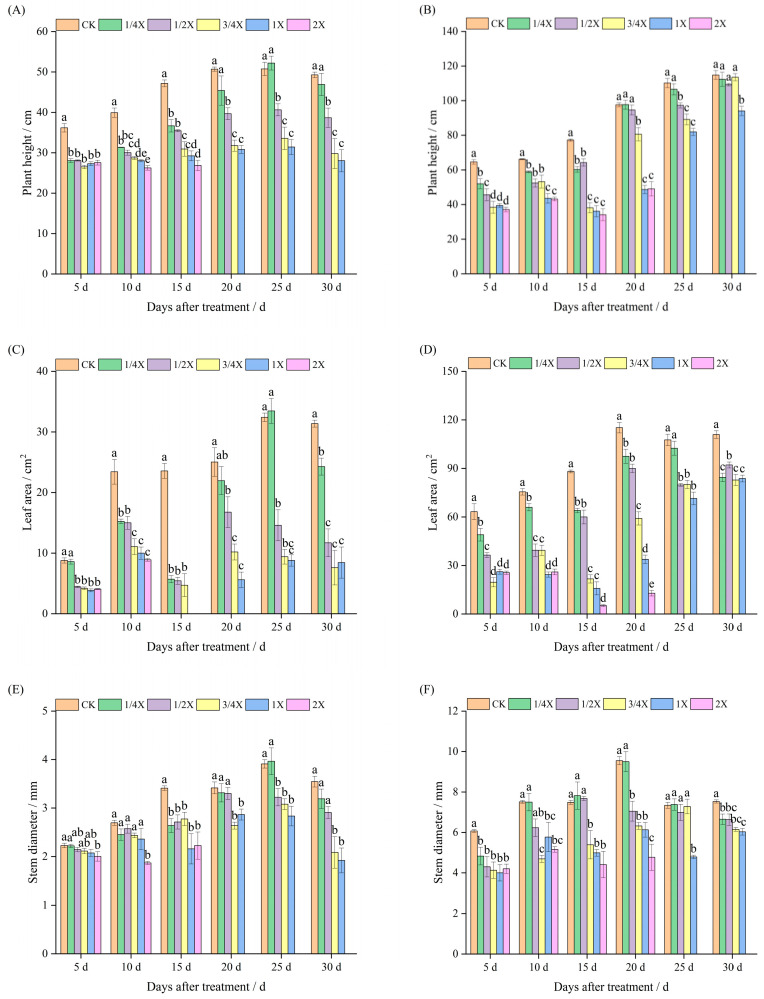
Effects of cyhalofop-butyl on agronomic traits. Figures (**A**,**C**,**E**) are pot experiments. Figures (**B**,**D**,**F**) are field experiments. Comparison between treatments of different concentrations on the same day, with lowercase letters representing a significant difference (*p* < 0.05).

It can be seen from Figure 1A that under indoor pot conditions, 5 days after Jingu 21 foxtail millet was treated with cyhalofop-butyl, the plant height of foxtail millet under each dose treatment was significantly lower than that of the control (CK). As the time after spraying extended, the inhibitory effect of cyhalofop-butyl on plant height gradually weakened. On the 20th day after spraying, all foxtail millet plants under the 2X (180 g a.i./hm^2^) treatment died. By the 30th day after spraying, the plant heights of foxtail millet under the 1/2X (45 g a.i./hm^2^), 3/4X (67.5 g a.i./hm^2^), and 1X (90 g a.i./hm^2^) treatments were still significantly lower than that of the control.

It can be seen from Figure 1C that 15 days after spraying, the inhibitory effect of cyhalofop-butyl on the leaf area of foxtail millet was the strongest. Among them, the foxtail millet under the 1X and 2X treatments was greatly affected by phytotoxicity, and the leaf area of the second top leaf could not be measured normally. On the 20th day after spraying, the foxtail millet under the 2X treatment died, and the leaf area of the foxtail millet under the 1X treatment began to recover growth. However, similar to the 1/2X and 3/4X treatments, the leaf area was still significantly lower than that of the control, which was 33.07%, 59.31%, and 77.60% lower than that of the control, respectively. On the 30th day after spraying, the leaf areas of foxtail millet under all dose treatments were significantly lower than that of the control.

It can be seen from Figure 1E that the effect of cyhalofop-butyl treatment on the stem diameter of foxtail millet was smaller than that on plant height and leaf area. However, as the time after spraying extended, the inhibitory effect on stem diameter gradually increased.

It can be seen from Figure 1B that under field conditions, the plant height of Jingu 21 foxtail millet decreased with the increase in cyhalofop-butyl dose. From the 5th to the 45th day after spraying, the plant height of foxtail millet under the 1X treatment was always significantly lower than that of the control. On the 30th day after spraying, all foxtail millet under the 2X treatment died. Except for the 1X treatment, there was no significant difference in plant height between other dose treatments and the control. However, on the 45th day after spraying, the plant heights of foxtail millet under all dose treatments were significantly lower than that of the control, and the inhibitory effect gradually increased.

It can be seen from Figure 1D,F that after cyhalofop-butyl treatment, the change trends of leaf area and stem diameter of Jingu 21 foxtail millet were similar to that of plant height, and the inhibitory effects on leaf area and stem diameter were stronger than that on plant height.

### 2.2. Effects of Spraying Different Doses of Cyhalofop-Butyl on Antioxidant Enzyme Activity of Foxtail Millet

**Figure 2:**
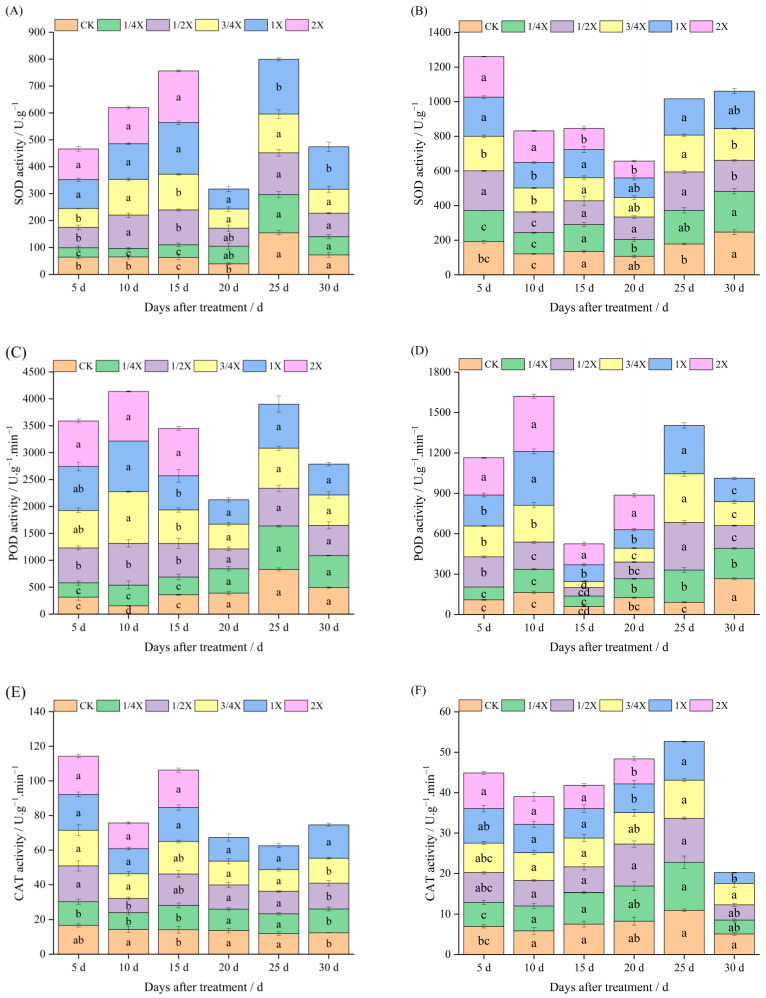
Effects of cyhalofop-butyl on antioxidant enzyme activity of foxtail millet. Figures (**A**,**C**,**E**) are pot experiments. Figures (**B**,**D**,**F**) are field experiments. Comparison between treatments of different concentrations on the same day, with lowercase letters representing a significant difference (*p* < 0.05).

It can be seen from Figure 2A that under pot conditions, the SOD activity in the leaves of Jingu 21 foxtail millet increased after cyhalofop-butyl treatment. On the 5th day after spraying, the SOD activity under the 1/4X (22.5 g a.i./hm^2^) treatment was significantly lower than that of the control, while the SOD activities under the 1X and 2X treatments were significantly higher than that of the control. On the 10th and 15th days after spraying, with the increase in spray dose, the SOD activity showed a trend of “first decreasing and then increasing”. Compared with the control, the SOD activities under the 1/2X, 3/4X, 1X, and 2X treatments increased significantly by 92.17%, 105.41%, 105.66%, 107.86% (on the 10th day after spraying) and 106.02%, 109.52%, 190.70%, 205.02% (on the 15th day after spraying), respectively, indicating that the herbicide stress on Jingu 21 foxtail millet was further strengthened during this stage. On the 20th day after spraying, the SOD activities under the 3/4X and 1X treatments were still significantly higher than that of the control, but the difference from the control was significantly smaller than that on the 15th day after spraying, decreasing from 109.52% and 190.70% to 80.75% and 89.59%, respectively. On the 25th day after spraying, only the SOD activity under the 1X treatment was significantly higher than that of the control, and there was no significant difference between other treatments and the control. On the 30th day after spraying, the SOD activity under the 2X treatment was still significantly higher than that of the control.

It can be seen from Figure 2C that after cyhalofop-butyl treatment, the POD activity in foxtail millet leaves increased significantly. The POD activity under the 1/4X treatment was lower than that of the control only on the 5th and 15th days after spraying, while the POD activities under other dose treatments were higher than that of the control from the 5th to the 15th day after spraying. On the 20th day after spraying, the foxtail millet under each treatment gradually recovered growth, and there was no significant difference in POD activity between the treatments and the control.

It can be seen from Figure 2E that from the 5th to the 15th day after spraying, the CAT activity in foxtail millet leaves showed a trend of “first decreasing and then increasing”. On the 20th day after spraying, the CAT activity recovered to a level with no significant difference from the control, indicating that the adaptability of foxtail millet to herbicide stress was enhanced.

Under field conditions, cyhalofop-butyl treatment also increased the antioxidant enzyme activities in foxtail millet leaves. It can be seen from Figure 2B that on the 5th day after spraying, the SOD activity in foxtail millet leaves under the 1/4X treatment was lower than that of the control, while the SOD activities under other dose treatments were higher than that of the control. On the 10th day after spraying, with the increase in spray dose, the SOD activity gradually increased. Among them, the SOD activities under the 3/4X, 1X, and 2X treatments were significantly higher than that of the control, increasing by 14.90%, 22.51%, and 50.80%, respectively, compared with the control. On the 15th day after spraying, the foxtail millet began to recover growth, and only the SOD activity under the 2X treatment was significantly lower than that of the control. On the 25th day after spraying, all foxtail millet under the 2X treatment died. On the 45th day after spraying, there was no significant difference in SOD activity between each dose treatment and the control.

It can be seen from Figure 2D that on the 5th and 10th days after spraying, the POD activity in foxtail millet leaves gradually increased with the increase in spray dose. The POD activities under the 3/4X, 1X, and 2X treatments were significantly higher than that of the control, increasing by 111.92%, 112.85%, 155.18% (on the 5th day after spraying) and 66.94%, 143.74%, 149.71% (on the 10th day after spraying), respectively, compared with the control. On the 45th day after spraying, the POD activities under all dose treatments were still significantly higher than that of the control.

It can be seen from Figure 2F that the CAT activity in foxtail millet leaves was less affected by cyhalofop-butyl. On the 5th day after spraying, only the CAT activity under the 2X treatment was significantly higher than that of the control. On the 30th day after spraying, the CAT activity under the 1X treatment was significantly lower than that of the control. In other time periods, there was no significant difference in CAT activity between each dose treatment and the control.

### 2.3. Effects of Spraying Different Doses of Cyhalofop-Butyl on MDA Content of Foxtail Millet

**Figure 3:**
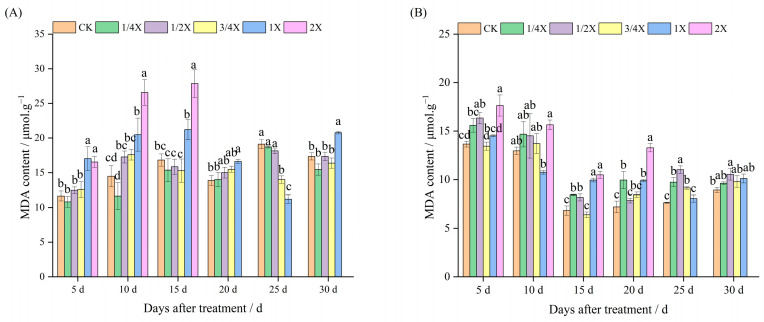
Effects of cyhalofop-butyl on MDA content of foxtail millet. (**A**) Pot experiment; (**B**) field experiment. Comparison between treatments of different concentrations on the same day, with lowercase letters representing a significant difference (*p* < 0.05).

It can be seen from Figure 3A that under pot conditions, the MDA content in the leaves of Jingu 21 foxtail millet increased after cyhalofop-butyl treatment. On the 5th and 10th days after spraying, with the increase in spray dose, the MDA content showed a trend of “first increasing and then decreasing”. From the 15th to the 20th day after spraying, the stress on foxtail millet weakened, but the MDA content under the 1X treatment was still significantly higher than that of the control by 26.17% and 19.75%, respectively. On the 30th day after spraying, only the MDA content under the 1X treatment was significantly higher than that of the control, and there was no significant difference between other dose treatments and the control.

It can be seen from Figure 3B that under field conditions, cyhalofop-butyl treatment also led to an increase in the MDA content in the leaves of Jingu 21 foxtail millet, indicating that the stress on foxtail millet was strengthened. From the 5th to the 20th day after spraying, the MDA content under the 2X treatment was significantly higher than that of the control. On the 25th day after spraying, the foxtail millet under the 2X treatment died. For other dose treatments, with the increase in spray dose, the MDA content showed a trend of “first increasing and then decreasing”. Among them, the MDA contents under the 1/4X, 1/2X, and 3/4X treatments were significantly higher than that of the control, increasing by 24.67%, 30.08%, and 30.08%, respectively, compared with the control. On the 30th day after spraying, only the MDA content under the 3/4X treatment was significantly higher than that of the control. On the 45th day after spraying, there was no significant difference in MDA content between each dose treatment and the control, indicating that the damage degree of the foxtail millet membrane system gradually recovered.

### 2.4. Study on the Weed Control Efficacy of Cyhalofop-Butyl in Foxtail Millet Fields

It can be seen from Table 1 that 5 days after spraying, with the increase in cyhalofop-butyl dose, the plant number control efficacy on *E. crus-galli,* a gramineous weed in foxtail millet fields, showed a trend of “first increasing and then decreasing”. The plant number control efficacy of each dose treatment on *E. crus-galli* reached more than 65%, and the fresh weight control efficacy reached more than 80%. Among them, the 1X treatment achieved 100% plant number control efficacy and fresh weight control efficacy on *E. crus-galli*. There were significant differences in the control efficacy of cyhalofop-butyl on different types of weeds. The plant number control efficacy on *D. sanguinalis* showed a trend of “first increasing and then decreasing”, while the fresh weight control efficacy showed a gradually increasing trend. Moreover, the plant number control efficacy and fresh weight control efficacy of each dose treatment on *D. sanguinalis* reached more than 80%. From the 10 to the 30 day after spraying, with the increase in spray dose, the plant number control efficacy of cyhalofop-butyl on *E. crus-galli* and *D. sanguinalis* gradually increased, and the high-dose treatments (1X, 2X) always maintained a high control effect.

## 3. Discussion

In modern agricultural production, the application of herbicides has completely changed the traditional weeding methods. However, while herbicides effectively control weeds, they may also cause phytotoxicity to crops and interfere with the normal growth and development of crops. As intuitive indicators reflecting the growth status of crops, agronomic traits can directly reflect the safety of herbicides to crops. Through the analysis of agronomic traits such as plant height, leaf area, biomass, and yield of crops, the safety of herbicides in crops can be proved [20]. The development, size, and area of leaves are important indicators for the growth, stress resistance, and yield formation of foxtail millet [21]. Herbicides have been widely proven to severely reduce plant growth and limit yield, including chlorotoluron [22], isoproturon [23], simetryne [24], and glyphosate [25]. Previous studies have found that pyrazosulfuron-methyl inhibits the growth of foxtail millet seedlings, manifested as a reduction in plant height, leaf area, and stem diameter [26].

The results of this study are consistent with the above previous research conclusions. With the increase in the dose of cyhalofop-butyl alone, the inhibitory effect on the plant height of Jingu 21 gradually increases. In both pot and field experiments, the Jingu 21 foxtail millet under the 2X treatment died in the later stage after spraying, while the foxtail millet under other dose treatments gradually recovered growth with the extension of time. It is worth noting that the phytotoxicity suffered by foxtail millet in the pot experiment is more severe than that in the field experiment. For example, 15 days after spraying in the pot experiment, the leaves of Jingu 21 under the 1X treatment broke due to phytotoxicity and could not be measured for leaf area. It was not until the 20th day after spraying that new second top leaves unfolded, and the foxtail millet gradually recovered growth. This may be related to the differences in substrate nutrient conditions, temperature and humidity regulation, and herbicide residue effects in the pot environment compared with the actual field environment.

When crops are under herbicide stress, herbicides, as exogenous substances, enter the crop body and trigger the activation of the crop’s reactive oxygen species scavenging system. This system protects cells from oxidative damage by scavenging free radicals and reactive oxygen species produced during metabolism. Malondialdehyde (MDA), as the main product of membrane lipid peroxidation [27], its content directly reflects the degree of plant cell membrane lipid peroxidation and can be used to measure the resistance of plants to external stress. CAT is an enzyme that mainly exists in peroxisomes and a small amount in the cytoplasm [28,29]. Many scholars have confirmed that under the action of herbicides, the activities of antioxidant enzymes including CAT will increase [24,30,31,32]. Studies have found that the content of malondialdehyde (MDA, a decomposition product of polyunsaturated fatty acid hydroperoxides, which has been widely used as a suitable biomarker to measure the degree of lipid peroxidation) increases after prometryne treatment, and this result can be used as an indicator of oxidative stress. The increase in MDA content indicates that the lipid peroxidation related to oxidative stress in the plant biofilm is enhanced [33]. Previously, Jiang and Yang [8] reported that the MDA content in the seedlings of common wheat (*Triticum aestivum*) increased under prometryne treatment. Similar results have been observed in many plant species treated with herbicides, and the reason is related to oxidative damage [34,35,36].

The results of this study are similar to the above conclusions. After cyhalofop-butyl treatment, the activities of SOD, POD, and CAT in foxtail millet all increased to varying degrees. As the time after spraying extended, the activities of antioxidant enzymes gradually decreased, indicating that foxtail millet responds to herbicide stress by regulating the antioxidant enzyme system, and as the stress time extends, foxtail millet gradually adapts to the stress environment. With the increase in spray dose, the MDA content shows a trend of “first decreasing and then increasing”.

At present, most studies on the effect of cyhalofop-butyl on weeds focus on *E.crus-galli*, *Leptochloa chinensis*, and other weeds in paddy fields [37,38], while there are relatively few studies on weeds in dry grain crop fields. This study is the first to systematically explore the control effect of cyhalofop-butyl on *E.crus-galli* and *D. sanguinalis* in foxtail millet fields and its safety on foxtail millet, filling the gap in the application research of cyhalofop-butyl in dry grain crop fields and providing a new technical option for chemical weed control in foxtail millet fields.

For ACCase inhibitors, besides cyhalofop-butyl evaluated in this study, other regionally applicable options include: 1. Fenoxaprop-p-ethyl: Effective against gramineous weeds (e.g., *E. crus-galli*, *D. sanguinalis*) and has been tested for safety in dryland crops like wheat in northern China. Its low-dose application (recommended 75–120 g a.i./hm^2^) may suit foxtail millet, though phytotoxicity risks to sensitive varieties require verification. 2. Clethodim: Broad-spectrum against annual/perennial grasses, with good environmental compatibility; it is currently used in dryland cash crops (e.g., soybeans) in the region, and preliminary trials suggest it could control foxtail millet field weeds if dose optimization (150–225 g a.i./hm^2^) and crop safety screening are conducted.

In subsequent studies, the following research can carry out the mixed experiment of cyhalofop-butyl and safe broad-leaved herbicides, and screen the mixed scheme of “expanding the weed control spectrum, reducing the dosage of single agent and reducing the phytotoxicity”, so as to build a safe weeding scheme with full coverage of “Gramineae+broad-leaved weeds” and provide more comprehensive technical support for chemical weeding in foxtail millet fields. At the same time, the scope of test varieties can be expanded to explore the safety differences in cyhalofop-butyl on different foxtail millet varieties, providing more comprehensive technical support for weed control in foxtail millet fields.

## 4. Materials and Methods

### 4.1. Materials

The test research base is located at the Crop Station of Shanxi Agricultural University (37°25′ N, 112°34′ E) with an altitude of 799.6 m. It belongs to a warm temperate continental climate, and the climatic conditions are suitable for carrying out foxtail millet-related experiments.

The tested foxtail millet variety is Jingu 21, a conventional variety, which was bred by the Economic Crop Research Institute of Shanxi Academy of Agricultural Sciences and has good adaptability and representativeness in the local area.

The tested herbicide is 10% cyhalofop-butyl aqueous emulsion, produced by Hebei Zhongbaolv Agricultural Crop Science and Technology Co., Ltd., Langfang, China with a production batch of 1805001. Other reagents used in the experiment are domestic analytical grade, ensuring the accuracy of the experiment and the reliability of the data.

Field experiment: The soil type is sandy loam. Before sowing, the basic fertility of the surface soil was determined (the determination results are shown in Table 2), and the determination work was completed by the Environmental Monitoring Center of Shanxi Agricultural University. The soil fertility status meets the requirements for foxtail millet growth and the experiment.

### 4.2. Experimental Design

#### 4.2.1. Study on the Safety of Different Doses of Cyhalofop-Butyl to Foxtail Millet

The experiment was conducted in the Crop Chemistry and Regulation Laboratory of Shanxi Agricultural University from March to December 2021. Danish Pindstrup substrate was filled into nutrient pots with a diameter of 7 cm × 7 cm and a depth of 7 cm, and the soil filling height was 3/4 of the nutrient pot. Plump and disease-free Jingu 21 seeds were selected and sown evenly in the nutrient pots, with 15–20 seeds per pot. After sowing, a layer of substrate about 1 cm thick was covered, and the substrate was saturated with water by means of bottom watering, then placed in a greenhouse with a temperature controlled at 25 °C for cultivation. When the foxtail millet grew to the 2–3 leaf stage, thinning was carried out, and 5 seedlings with consistent growth were retained in each pot. When the foxtail millet reached the 3–5 leaf stage, according to the test dose settings shown in Table 3, the 3WP-2000 self-propelled spray tower produced by Nanjing Agricultural Machinery Research Institute was used for foliar spray treatment on the foxtail millet. The greenhouse conditions during the entire cultivation period were controlled as follows: photoperiod of 16 h light/8 h dark; illuminated intensity of 12,000 lx. Each treatment was set with 5 repetitions, and clear water treatment was used as the blank control (CK) at the same time. After the spray treatment, the nutrient pots were continued to be cultured in the greenhouse, and regular water supply was carried out by means of bottom irrigation. The growth status and symptom changes in the plants were closely observed, and foxtail millet plants with consistent growth were selected on the 5, 10, 15, 20, 25, 30, and 45 days after herbicide application to determine the relevant indicators.

The experiment was carried out in the experimental field of the Crop Station of Shanxi Agricultural University from May to October 2021. Sowing was conducted in May 2021. 1–2 days before sowing, compound fertilizer was applied at 750 kg/hm^2^ as base fertilizer, followed by soil preparation. No additional fertilizer was applied during the growth period of foxtail millet, and only conventional field management such as weeding and irrigation was carried out. A randomized block design was adopted in the experiment, with different doses of 10% cyhalofop-butyl aqueous emulsion set as treatments, and clear water treatment as the control (CK). Each plot had an area of 30 m^2^ (5 m × 6 m), and each treatment was set with 3 repetitions. When the foxtail millet grew to the 3–5 leaf stage, cyhalofop-butyl was sprayed according to the doses shown in Table 3.

#### 4.2.2. Study on the Weed Control Efficacy of Different Doses of Cyhalofop-Butyl in Foxtail Millet Fields

The experiment was carried out at the Dingxiang Experimental Base of Shanxi Agricultural University from May to September 2021. A randomized block design was adopted. Cyhalofop-butyl with different doses was sprayed when the foxtail millet was at the 3–5 leaf stage (the dose settings are shown in Table 3), and the control treatment was sprayed with the same amount of clear water (CK). Each treatment was set with 3 repetitions, and the plot area was 30 m^2^ (6 m × 5 m). The weed control efficacy of each plot was investigated on the 5th, 10th, 15th, 20th, 25th, and 30th days after herbicide application. The 5-point sampling method was used for fixed-point investigation, with an investigation area of 0.25 m^2^ (0.5 m × 0.5 m) at each point. The specific distribution of the 5-point sampling method in this experiment is “diagonal 5-point sampling method”: in a 30-m residential area (6 m long and 5 m wide), five sampling points are evenly arranged along two diagonals (from the upper left corner to the lower right corner and from the upper right corner to the lower left corner), and one of them is located at the intersection of the two diagonals (the center of the residential area). The other four points are located at 1/4 and 3/4 of the diagonal (1.5 m and 4.5 m away from the edge of the plot), and each sampling point is a square area of 0.5 m × 0.5 m, which ensures that the sampling covers different positions of the plot and reduces the influence of edge effect and uneven weed distribution on the data.

The number of weeds in each treatment plot was recorded, and the fresh weight of weeds was measured. The plant number control efficacy and fresh weight control efficacy of weeds were calculated, respectively, using the following formulas:

Weed plant number (fresh weight) control efficacy (%) = [(Number/fresh weight of weeds in blank control plot-Number/fresh weight of weeds in treatment plot)/Number/fresh weight of weeds in blank control plot] × 100.

### 4.3. Analysis of Test Indicators and Methods

Foxtail millet samples were collected on the 5th, 10th, 15th, 20th, 25th, 30th, and 45th days after herbicide application. The functional leaves of the second top leaf and the complete plants of foxtail millet were selected. Three repeated samples were collected from each plot, and the samples were placed in an ice box and quickly brought back to the laboratory. Among them, the plant samples were used to determine the agronomic traits, and the remaining leaf samples were stored in a −80 °C refrigerator for the subsequent determination of antioxidant enzyme activities and MDA content.

An amount of 0.1 g (accurate to 0.0001 g) of the second top leaf of foxtail millet under different treatments was weighed and added to a mortar containing 2 mL of phosphate buffer (pH 7.8) for thorough grinding, and the grinding was washed twice. The mixture was transferred to a 10 mL centrifuge tube. Centrifugation was carried out using a high-speed refrigerated centrifuge (model: Sigma 3-18KS, Sigma Laborzentrifugen GmbH, Osterode am Harz, Germany) with a swing-out rotor (model: 19776-H, max load: 24 × 5 g) at 4 °C and 12,000 rpm for 15 min, and the supernatant was the enzyme extract, which was used for the determination of SOD, POD, and CAT activities.

SOD activity determination: The activity of SOD was determined by the nitroblue tetrazolium (NBT) photochemical reduction method [39]. The reaction system (3 mL) contained 50 mmol/L phosphate buffer (pH 7.8), 13 mmol/L methionine, 75 μmol/L NBT, 10 μmol/L EDTA-Na_2_, 2 μmol/L riboflavin, and 0.1 mL enzyme extract. The control group was set with 0.1 mL phosphate buffer (pH 7.8) instead of the enzyme extract. The reaction was initiated by placing the reaction tubes under a white fluorescent lamp (20 W, light intensity: 4000 lx) at 25 °C for 20 min, and then the light was turned off to terminate the reaction. The absorbance value was measured at 560 nm using a spectrophotometer (Shunyu Henping Instrument, LLC, Shanghai, China). One unit of SOD activity was defined as the amount of enzyme required to inhibit 50% of NBT photochemical reduction, and the activity was expressed as U/g.

POD activity determination: The activity of POD was determined by the guaiacol oxidation method [40]. The reaction system (3 mL) included 50 mmol/L phosphate buffer (pH 6.0), 20 mmol/L guaiacol, 40 mmol/L H_2_O_2_, and 0.05 mL enzyme extract. The increase in absorbance at 470 nm was recorded continuously for 3 min at 25 °C using a spectrophotometer. One unit of POD activity was defined as the amount of enzyme that causes an increase of 0.01 in absorbance per minute, and the activity was expressed as U/(g·min).

CAT activity determination: The activity of CAT was determined by the hydrogen peroxide method [41]. The reaction system (3 mL) consisted of 50 mmol/L phosphate buffer (pH 7.0), 10 mmol/L H_2_O_2_, and 0.1 mL enzyme extract. The decrease in absorbance at 240 nm was measured continuously for 3 min at 25 °C using a spectrophotometer. One unit of CAT activity was defined as the amount of enzyme that decomposes 1 μmol of H_2_O_2_ per minute, and the activity was expressed as U/(g·min).

The MDA content was determined by the thiobarbituric acid (TCA) method [42]. First, 0.4 g of the second top leaf of foxtail millet was weighed, crushed with a sample grinder, and transferred to a centrifuge tube. Then, 5 mL of 1% TCA solution and 5 mL of 0.5% TCA solution were added, and the mixture was fully shaken and then heated in a boiling water bath for 15 min. Then, it was quickly taken out and cooled to room temperature in cold water. The cooled sample was centrifuged using a high-speed refrigerated centrifuge (model: Sigma 3-18KS, Sigma Laborzentrifugen GmbH, Osterode am Harz, Germany) with a swing-out rotor (model: 19776-H, max load: 24 × 5 g) at 4 °C and 3000 rpm for 15 min. The supernatant was taken and its volume was recorded. Using 0.5% TCA solution as a reference, the absorbance values of the supernatant at 532 nm and 600 nm wavelengths were measured, respectively, and the MDA content was calculated based on this.

### 4.4. Data Processing

Excel 2021 (Microsoft, Redmond, WA, USA) software was used for preliminary statistics and sorting of the experimental data; SPSS 23.0 (IBM Corporation, Armonk, NY, USA) software was used for Duncan’s new multiple range test for significance analysis, with the significance level set at *p* < 0.05; Origin 2021 (OriginLab, Northampton, MA, USA) software was used to draw experimental charts to ensure the intuitiveness and accuracy of data presentation.

## 5. Conclusions

Cyhalofop-butyl alone can inhibit the normal expression of the agronomic traits of Jingu 21 foxtail millet to a certain extent, but with the extension of time after spraying, the growth of foxtail millet gradually recovers. Among them, the 2X treatment causes severe phytotoxicity to Jingu 21, and the foxtail millet cannot recover growth and eventually dies. In terms of weed control efficacy, the control effects of the 1/4X, 1/2X, and 3/4X treatments on weeds in foxtail millet fields gradually increase with the increase in dose, and the 1X treatment achieves the best weed control effect. Comprehensively considering the safety of foxtail millet and the weed control efficacy, it can be initially determined that the 1/4X (22.5 g a.i./hm^2^), 1/2X (45 g a.i./hm^2^), and 3/4X (67.5 g a.i./hm^2^) treatments are safe for Jingu 21. On the premise of ensuring the weed control effect, the 1/2X (45 g a.i./hm^2^) and 3/4X (67.5 g a.i./hm^2^) treatments can be preferentially applied to weed control in Jingu 21 foxtail millet fields.

## Figures and Tables

**Table 1:** Control effects of cyhalofop-butyl on gramineous weeds in foxtail millet field.

Days After Treatment	Treatment	*E. crus-galli*				*D. sanguinalis*			
		Number of Plants/m^2^	Plant Control Effect/%	Fresh Weight/g	Fresh Weight Control Effect/%	Number of Plants/m^2^	Plant Control Effect/%	Fresh Weight/g	Fresh Weight Control Effect/%
5 d	CK	7.75 a	/	41.85 a	/	7.33 a	/	2.08 a	/
	1/4X	2.50 b	67.74 b	6.59 b	84.25 b	2.00 b	72.73 a	0.32 b	84.77 a
	1/2X	0.50 b	93.55 ab	1.59 b	96.21 ab	1.75 b	76.14 a	0.28 b	86.45 a
	3/4X	0.50 b	93.55 ab	1.64 b	96.09 ab	1.50 b	79.55 a	0.03 b	98.38 a
	1X	0.00 b	100.00 a	0.00 b	100.00 a	1.25 b	82.95 a	0.03 b	98.64 a
	2X	0.25 b	96.78 a	0.59 b	98.59 ab	1.00 b	86.36 a	0.04 b	98.28 a
10 d	CK	6.50 a	/	98.66 a	/	2.00 a	/	16.00 a	/
	1/4X	4.00 b	38.46 c	39.61 b	59.85 b	1.00 b	57.14 b	4.53 b	71.72 a
	1/2X	2.50 bc	61.54 b	24.27 b	75.40 ab	0.25 bc	89.29 ab	3.14 b	80.37 a
	3/4X	1.75 cd	73.08 ab	12.98 b	86.85 a	0.50 bc	78.57 ab	4.00 b	75.00 a
	1X	0.50 d	92.31 a	5.11 b	94.82 a	0.00 c	100.00 a	0.00 b	100.00 a
	2X	0.50 d	92.31 a	4.29 b	95.65 a	0.00 c	100.00 a	0.00 b	100.00 a
15 d	CK	8.00 a	/	315.46 a	/	4.50 a	/	44.18 a	/
	1/4X	4.75 ab	40.63 b	96.54 ab	69.40 b	0.50 b	88.89 a	1.28 b	97.10 a
	1/2X	4.00 ab	50.00 ab	93.63 ab	70.32 b	0.25 b	94.44 a	1.72 b	96.11 a
	3/4X	2.25 b	71.88 ab	42.41 b	86.56 ab	0.00 b	100.00 a	0.00 b	100.00 a
	1X	0.25 b	96.88 a	0.30 b	99.90 a	0.00 b	100.00 a	0.00 b	100.00 a
	2X	0.25 b	96.88 a	20.75 b	93.42 a	0.00 b	100.00 a	0.00 b	100.00 a
20 d	CK	5.00 a	/	202.27 a	/	3.33 a	/	3.57 a	/
	1/4X	4.00 ab	46.67 b	155.37 ab	76.19 b	1.25 b	62.50 b	0.59 b	83.55 b
	1/2X	2.75 abc	45.00 b	47.01 bc	76.76 b	1.25 b	62.50 b	0.27 b	92.37 ab
	3/4X	1.75 bcd	65.00 ab	10.50 bc	94.81 a	1.00 b	70.00 ab	0.37 b	89.65 ab
	1X	0.50 cd	90.00 a	9.00 bc	95.55 a	0.25 b	92.50 ab	0.14 b	96.22 ab
	2X	0.00 d	100.00 a	0.00 c	100.00 a	0.00 b	100.00 a	0.00 b	100.00 a
25 d	CK	6.75 a	/	405.25 a	/	3.33 a	/	35.67 a	/
	1/4X	2.75 b	59.26 b	100.25 b	75.26 b	1.00 b	70.00 a	3.25 b	90.89 a
	1/2X	2.50 b	62.96 b	99.25 b	75.51 b	0.75 b	77.50 a	1.42 b	96.03 a
	3/4X	2.50 b	62.96 b	53.25 b	86.86 ab	0.75 b	77.50 a	1.50 b	95.79 a
	1X	0.75 b	88.89 a	39.50 b	90.25 ab	0.00 b	100.00 a	0.00 b	100.00 a
	2X	0.75 b	88.89 a	15.50 b	96.18 a	0.00 b	100.00 a	0.00 b	100.00 a
30 d	CK	7.50 a	/	485.25 a	/	3.67 a	/	58.67 a	/
	1/4X	3.00 b	60.00 b	156.25 b	67.80 c	2.50 ab	31.82 c	29.75 bc	49.29 b
	1/2X	3.00 b	60.00 b	139.50 b	71.25 bc	1.25 bc	65.91 b	16.75 bc	71.45 ab
	3/4X	2.25 bc	70.00 ab	41.75 b	91.40 ab	1.00 bc	72.73 ab	11.19 bc	80.92 a
	1X	0.50 bc	93.33 a	2.00 b	99.59 a	0.00 c	100.00 a	0.00 c	100.00 a
	2X	0.25 c	96.67 a	0.75 b	99.85 a	0.00 c	100.00 a	0.00 c	100.00 a

Note: Comparison between treatments of different concentrations on the same day, with lowercase letters representing a significant difference (*p* < 0.05). The ‘Number of Plants/m^2^′ refers to the number of target weed plants counted per square meter in the sampling area (0.25 m^2^ per sampling point, converted to per square meter).

**Table 2:** Basic fertility of soil tested.

Years	pH	Available K mg/kg	Olsen P mg/kg	Available N mg/kg	Total N g/kg	Total P g/kg	Total K g/kg	Organic g/kg
2021	8.20	291.09	23.62	51.84	1.04	1.12	18.75	23.36

**Table 3:** Setting scheme of different doses of cyhalofop-butyl/g a.i./hm^2^.

Treatment	1/4X	1/2X	3/4X	1X	2X	CK
Spraying amount	22.5	45	67.5	90	180	0

Note: There commended dose of cyhalofop-butyl is 900 mL/hm^2^, and the water content is 525 L/hm^2^.

## Data Availability

The data that support this study are available upon reasonable request from the corresponding author. The data are not publicly available due to privacy.

## References

[1] Hwang J.I., Norsworthy J.K., McElroy J.S., Rutland C.A., Barber L.T., Butts T.R. (2023). Metabolic exploration for cyhalofop-butyl antagonism in Barnyardgrass [*Echinochloa crus-galli* (L.) P. Beauv.] following pretreatment of malathion. J. Agric. Food Chem..

[2] Wang L., Sun X., Peng Y., Chen K., Wu S., Guo Y., Zhang J., Yang H., Jin T., Wu L. (2022). Genomic insights into the origin, adaptive evolution, and herbicide resistance of *Leptochloa chinensis*, a devastating tetraploid weedy grass in rice fields. Mol. Plant.

[3] Cutti L., Rigon C.A.G., Girelli N., Angonese P.S., Ulguim A.D.R., Merotto A. (2022). The safener isoxadifen-ethyl confers fenoxaprop-p-ethyl resistance on a biotype of *Echinochloa crus-galli*. Pest Manag. Sci..

[4] Ore A., Olayinka E.T. (2019). Toxic Mechanisms of Aryloxyphenoxypropionates in Target and Nontarget Organisms. Int. J. Biochem. Res. Rev..

[5] Zhang J., Weaver S.E., Hamill A. (2000). Risks and reliability of using herbicides at below-labeled doses. Weed Technol..

[6] Robinson A.P., Simpson D.M., Johnson W.G. (2013). Response of glyphosate-tolerant soybean yield components to dicamba exposure. Weed Sci..

[7] Boulahia K., Ould said C., Abrous-Belbachir O. (2023). Exogenous application of salicylic acid improve growth and some physio-biochemical parameters in herbicide stressed *Phaseolus vulgaris* L. Gesunde Pflanzen..

[8] Jiang L., Yang H. (2009). Prometryne-induced oxidative stress and impact on antioxidant enzymes in wheat. Ecotoxicol. Environ. Saf..

[9] Jiang L., Maa L., Sui Y., Han S.Q., Wu Z.Y., Feng Y.X., Yang H. (2010). Effect of manure compost on the herbicide prometryne bioavailability to wheat plants. J. Hazard. Mater..

[10] Singh G., Wright D. (1999). Effects of herbicides on nodulation, symbiotic nitrogen fixation, growth and yield of pea (*Pisum sativum*). J. Agric. Sci..

[11] Boulahia K., Carol P., Planchais S., Abrous-Belbachir O. (2016). *Phaseolus vulgaris* L. seedlings exposed to prometryn herbicide contaminated soil trigger an oxidative stress response. J. Agric. Food Chem..

[12] Kehrer J.P. (1993). Free radicals as mediators of tissue injury and disease. Crit. Rev. Toxicol..

[13] Chen J., Shiyab S., Han F.X., Monts D.L., Waggoner C.A., Yang Z., Su Y. (2009). Bioaccumulation and physiological effects of mercury in *Pteris vittata* and *Nephrolepis exaltata*. Ecotoxicology.

[14] Mahaboob Khan S., Kour G. (2007). Subacute oral toxicity of chlorpyriphos and protective effect of green tea extract. Pestici. Biochem. Physiol..

[15] Panfili I., Bartucca M.L., Marrollo G., Povero G., Del Buono D. (2019). Application of a plant biostimulant to improve maize (*Zea mays*) tolerance to metolachlor. J. Agric. Food Chem..

[16] Qu Q., Li Y., Zhang Z.Y., Cui H.Z., Zhao Q.Q., Liu W.Y., Lu T., Qian H.F. (2021). Effects of S-metolachlor on wheat (*Triticum aestivum* L.) seedling root exudates and the rhizosphere microbiome. J. Hazard. Mater..

[17] Bowler C., Slooten L., Vandenbranden S., De Rycke R., Botterman J., Sybesma C., Van Montagu M., Inzé D. (1991). Manganese superoxide dismutase can reduce cellular damage mediated by oxygen radicals in transgenic plants. EMBO J..

[18] McKersie B., Chen Y., Beus M., Bowley S., Bowler C., Inze D., D’Halluin K., Botterman J. (1993). Superoxide dismutase enhances tolerance of Freezing stress in transgenic alfalfa (*Medicao saiva* L.). Plant Physiol..

[19] Wang M.E., Zhou Q.X. (2006). Effects of herbicide chlorimuron-ethyl on physiological mechanisms in wheat (*Triticum aestivum*). Ecotoxicol. Environ. Saf..

[20] Yuan X.Y., Guo P.Y., Qi X., Ning N., Wang H., Wang H.F., Wang X., Yang Y. (2013). Safety of herbicide sigma broad on radix isatidis (*Isatis indigotica* fort.) seedlings and their photosynthetic physiological responses. Pestic. Biochem. Phys..

[21] Aidoo M.K., Bdolach E., Fait A., Lazarovitch N., Rachmilevitch S. (2016). Tolerance to high soil temperature in foxtail millet (*Setaria italica* L.) is related to shoot and root growth and metabolism. Plant Physiol. Bioch..

[22] Song N.H., Yin X.L., Chen G.F., Yang H. (2007). Biological responses of wheat (*Triticum aestivum*) plants to the herbicide chlorotoluron in soils. Chemosphere.

[23] Liang L., Lu Y.L., Yang H. (2012). Toxicology of isoproturon to the food crop wheat as affected by salicylic acid. Environ. Sci. Pollt. Res. Int..

[24] Jiang L., Yang Y., Jia L.X., Lin J.L., Liu Y., Pan B., Lin Y. (2016). Biological responses of wheat (*Triticum aestivum*) plants to the herbicide simetryne in soils. Ecotoxicol. Environ. Saf..

[25] Fernandes B., Soares C., Braga C., Rebotim A., Rafael Ferreira R., Ferreira J., Fidalgo F., Pereira R., Cachada A. (2020). Ecotoxicological assessment of a glyphosate-based herbicide in cover plants: *Medicago sativa* L. as a model species. Appl Sci..

[26] Ma K., Zhang W., Zhang L., He X., Fan Y., Alam S., Yuan X. (2021). Effect of pyrazosulfuron-methyl on the photosynthetic characteristics and antioxidant systems of foxtail millet. Front. Plant Sci..

[27] Jalal A., Oliveira Junior J.C., Ribeiro J.S., Fernandes G.C., Mariano G.G., Trindade V.D.R., Reis A.R.D. (2020). Hormesis in plants: Physiological and biochemical responses. Ecotoxicol. Environ. Saf..

[28] Foyer C.H., Noctor G. (2000). Oxygen processing in photosynthesis regulation and signaling. New Phytol..

[29] Arora A., Sairam R.K., Srivastava G.C. (2002). Oxidative stress and antioxidative system in plants. Curr. Sci..

[30] Cui J., Zhang R., Wu G.L., Zhu H.M., Yang H. (2010). Salicylic acid reduces napropamide toxicity by preventing its accumulation in rapeseed (*Brassica napus* L.). Arch. Environ. Contam. Toxicol..

[31] Spormann S., Soares C., Fidalgo F. (2019). Salicylic acid alleviates glyphosate-induced oxidative stress in *Hordeum vulgare* L.. J. Environ. Manag..

[32] Ould said C., Boulahia K., Eid M.A.M., Rady M.M., Djebbar R.R., Abrous-Belbachir O. (2021). Exogenously used proline offers pPotent antioxidative and osmoprotective strategies to re-balance growth and physio-biochemical attributes in herbicide-stressed *Trigonella foenum-graecum*. J. Soil Sci. Plant Nutr..

[33] Wang Q., Que X., Zheng R., Pang Z., Li C., Xiao B. (2015). Phytotoxicity assessment of atrazine on growth and physiology of three emergent plants. Environ. Sci. Pollut. Res..

[34] Shopova E., Brankova L., Katerova Z., Dimitrova L., Todorova D., Sergiev I., Talaat N.B. (2021). Salicylic Acid Pretreatment Modulates Wheat Responses to Glyphosate. Crops.

[35] Yu Q.Q., Lu F.F., Ma L.Y., Yang H., Song N.H. (2021). Residues of reduced herbicides terbuthylazine, ametryn, and atrazine and toxicology to maize and the environment through salicylic acid. ACS Omega.

[36] Li X., Riaz M., Song B., Liang X., Liu H. (2022). Exogenous salicylic acid alleviates fomesafen toxicity by improving photosynthetic characteristics and antioxidant defense system in sugar beet. Ecotoxicol. Environ. Saf..

[37] Ntanos D.A., Koutroubas S.D., Mavrotas C. (2000). Barnyardgrass (*Echinochloa crus-galli*) control in water-seeded rice (*Oryza sativa*) with cyhalofopbutyl. Weed Technol..

[38] Deng W., Cai J., Zhang J., Chen Y., Chen Y., Di Y., Yuan S. (2019). Molecular basis of resistance to ACCase-inhibiting herbicide cyhalofop-butyl in Chinese sprangletop (*Leptochloa chinensis* (L.) Nees) from China. Pestic. Biochem. Physiol..

[39] Beauchamp C., Fridovich I. (1971). Superoxide dismutase: Improved assays and an assay applicable to acrylamide gels. Anal. Biochem..

[40] Chance B., Maehly A.C. (1955). Assay of catalases and peroxidases. Methods Enzymol..

[41] Aebi H. (1984). Catalase in vitro. Methods Enzymol..

[42] Tsikas D. (2017). Assessment of lipid peroxidation by measuring malondialdehyde (MDA) and relatives in biological samples: Analytical and biological challenges. Anal. Biochem..

